# Social and Poor-Law Problems

**Published:** 1907-07-13

**Authors:** 


### SOCIAL AND POOR-LAW PROBLEMS.

### THE AFTER-CARE OF THE INSANE.

A Society doing a good work and little regarded by the public, is the After-care Association; it is interested in poor persons discharged as recovered from asylums for the insane. A lunatic may be entirely recovered, and yet not be immediately able to go back to his old environment, where the responsibilities and anxieties which perhaps had before helped to shake the mind, at once re-establish their influence. In such cases a change of scene and air, a quiet time without the obvious contiol exercised in the asylum and free from the troubles of ordinary life, may make all the difference between a relapse and a permanent recovery. Among the poor there are also many cases where the patient has lost his situation through his malady, and in such instances the association strives to find suitable employment, and gives pecuniary help till it is found. Clothing, also, and tools are provided when necessary. In dubious cases the patients may be boarded out in the country under proper care, or placed in suitable institutions until a more permanent provision can be made for them. In all cases the idea is to keep the mind at ease until it is once more habituated to the responsibilities of ordinary life. The results of the work of the association are excellent, and it has been the means of placing on a footing of self-support many who were destitute and friendless when they left the asylum. The beneficiaries are drawn from more than one class, and to find suitable homes and employment for all is a delicate as well as a difficult task; but the results fully justify the effort. Subscriptions to this useful society may be sent to the Secretary, H. Thornhill Roxby, Esq., Church House, Dean's Yard, Westminster, S.W.

### THE PAROCHIAL MISSION-WOMEN'S ASSOCIATION.

A very useful, though modest, work is done by the Parochial Mission-Women's Association. These women are drawn from the working class, and visit the poor in their homes as friends and equals. They go empty-handed. Their own salary averages only 13s. weekly, and they have no alms entrusted to them by the Association. Their special mission is to bring home to the poor and thriftless, even to the idle and degraded, the lesson of self-help. The mission-woman begins by making friends with those around her; then, when acquaintance has progressed a little, she produces a deposit-card, and offers to take care of any money —pence or half-pence—that can be spared for a rainy day. There is no interest promised on the deposit, no bribe to induce saving; only the promise that when the money is wanted for real need it will be forthcoming, and that it can be returned in the shape of necessaries of good quality at store prices—a very different quality and price from what the poor as a rule can obtain for themselves. There is often difficulty in getting the card taken, but when the odd coppers begin to return in such solid form as just mentioned it both seems like a gift, and yet brings with it the encouraging thought that the beneficiary has earned it all. The taint of charity, often so demoralising, is absent, and from the time a poor woman begins to deposit her pence with the mission-women there often dates a marvellous revival in the family circumstances. The mission-woman is not a teacher of religion, but she must be a Christian woman, who, living her own honourable and helpful life by the grace of God, proves by that life the power of true religion. Mission-women are placed only in parishes where the incumbent desires their presence, so that they are in no way intruders into the domains of others, but in an unpretentious way are often a clergyman's best helpers, all the more efficient because they belong to the class among whom they labour, and whose ideas, ambitions, and temptations they can the better understand and sympathise with. It is nearly fifty years since the Association was founded, and those who first supported it have passed away. The first Countess of Selborne was one of the founders of the mission, and her daughter, Lady Sophia Palmer, has recently written a very interesting account of it. A mission can be supported for a year for £36, which shows how economical the scheme is. The clergy appreciate the work of the mission-women greatly, and appeals for their help are often received, but for lack of funds they cannot always be responded to. And while funds are dwindling, the necessity for the work in the slums of London is ever increasing. Therefore subscriptions or donations will be gladly received by the Secretary, at the office of the Parochial Mission-Women's Association, Church House, Dean's Yard, Westminster, or by the Treasurer, the Earl Waldegrave.

### THE PROBLEM OF THE ABLE-BODIED POOR.

The attention of the Local Government Board has been lately drawn to the fact that the number of able-bodied men in workhouses tends to increase. Pauperism as a whole has increased largely of recent years. This has been due partly to bad trade, but partly, also, to the better treatment of the pauper class both in and out of the workhouse, and the recent improvement of affairs in the labour market has not affected—so far as Poor-law statistics go—the very class who ought to have benefited, the able-bodied men. In a parish in Birmingham where the population shows a tendency to decrease, the number of indoor poor continues to increase, and the increase is most marked among the men. This regrettable state of affairs is largely due to want of proper classification. Where young and old, healthy and infirm, are all placed together, common humanity bids those in authority treat the aged with a certain amount of consideration, and the others benefit by the leniency. The long-continued talk about old-age pensions has largely affected the sentiment of Poor-law administration, and Guardians in many cases are inclined to go as far as the law will allow them in making relief as pleasant and non-humiliating as possible; with the old this may be permissible, but unfortunately the young and lazy at present also profit by the kindliness shown. Similarly, a period of trade depression which drives people who have hitherto been self-supporting to seek Poor-law relief, softens the hearts of Guardians, and they make the conditions as little disagreeable as possible for this class. But, cruel as it may seem to say so, this is unwise; for having once found that the workhouse is not such a bad place as they had fancied, these people lose the very self-respect which led to their being kindly treated. They degenerate into habitual paupers, ready to go into the house on the least excuse. The only remedy is to make workhouse conditions harder for the able-bodied than for those of the same class outside (and this is what in theory they are supposed by the law to be), while dealing as kindly as may be with the old and infirm, especially with those who can show that they have lived honestly and honourably until old age or sickness came upon them. The recent "strike" at the colony for the unemployed at South Ockenden—due to the fact that the men were expected to work as long as the agricultural labourers into which it is meant to transform them, although they are much better fed than these—shows how far from the disciplinary ideal of the workhouse present-day Poor-law administration has moved, and the result is not difficult to guess. So long as the able-bodied pauper fares better than the independent worker of the same class, so long will our workhouses and so-called labour colonies be filled with men who, though in good health, say they cannot find work.

